# Severe atypical juvenile pityriasis rubra pilaris diagnosed in adulthood with a dramatic improvement with ustekinumab

**DOI:** 10.1002/ski2.389

**Published:** 2024-04-11

**Authors:** Rebecca L. McCarthy, Jaimie Oldham, Elsa Barbosa, Catriona Sinclair, Malvina Cunningham, Edel A. O’Toole

**Affiliations:** ^1^ Faculty of Medicine and Dentistry Centre for Cell Biology and Cutaneous Research Blizard Institute Queen Mary University of London London UK; ^2^ Department of Dermatology Royal London Hospital Barts Health NHS Trust London UK; ^3^ Department of Dermatology Broomfield Hospital Mid and South Essex NHS Foundation Trust Basildon UK

## Abstract

Pityriasis rubra pilaris (PRP) is a rare skin disease which manifests as a psoriasiform dermatosis and palmoplantar keratoderma and has significant clinical‐histopathological overlap with psoriasis. Recently, several case reports have demonstrated successful treatment of PRP with anti‐IL7A and anti‐IL12/anti‐IL23 monoclonal antibodies. We report a case of atypical juvenile PRP definitively diagnosed during adulthood with presence of CARD14 mutation. This case demonstrates a dramatic improvement with ustekinumab and highlights the role of genetic testing in chronic disease of diagnostic uncertainty.

## Graphical abstract

Pityriasis rubra pilaris (PRP) is a rare skin disease which manifests as a psoriasiform dermatosis and palmoplantar keratoderma and has significant clinical‐histopathological overlap with psoriasis. We report a case of atypical juvenile PRP definitively diagnosed during adulthood with presence of CARD14 mutation. This case demonstrates a dramatic improvement with ustekinumab and highlights the role of genetic testing in chronic disease of diagnostic uncertainty.

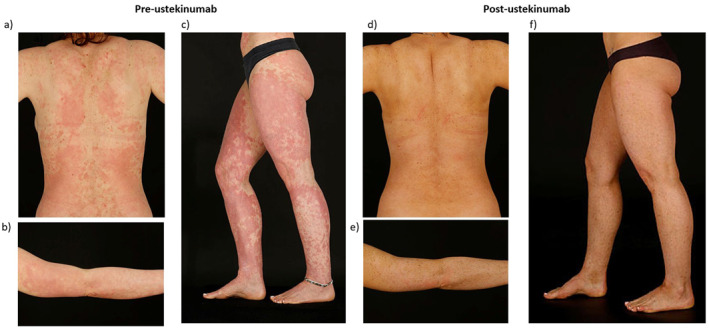

## 1 INTRODUCTION

Pityriasis rubra pilaris (PRP) is a rare skin disease which manifests as a psoriasiform dermatosis and palmoplantar keratoderma, with significant clinical‐histopathological overlap with psoriasis. There are six PRP subtypes classified by age at onset, disease extent, prognosis, and other features.^1^ We present a case of atypical juvenile onset (type V) PRP with a dramatic improvement with ustekinumab. We obtained written informed consent for the use of the patient's anonymised clinical information and images.

The patient is a 32‐year‐old female, known to dermatology departments since birth. From aged six weeks and throughout childhood she had scaly erythroderma, sparing the flexural surfaces and parts of the back, and yellowish hyperkeratotic palms and soles. She was initially thought to have congenital ichthyosiform erythroderma and was subsequently diagnosed with progressive symmetrical erythrokeratoderma. She was a non‐smoker with no other health conditions or medications. Her paternal grandmother had ichthyosis during childhood.

**FIGURE 1:**
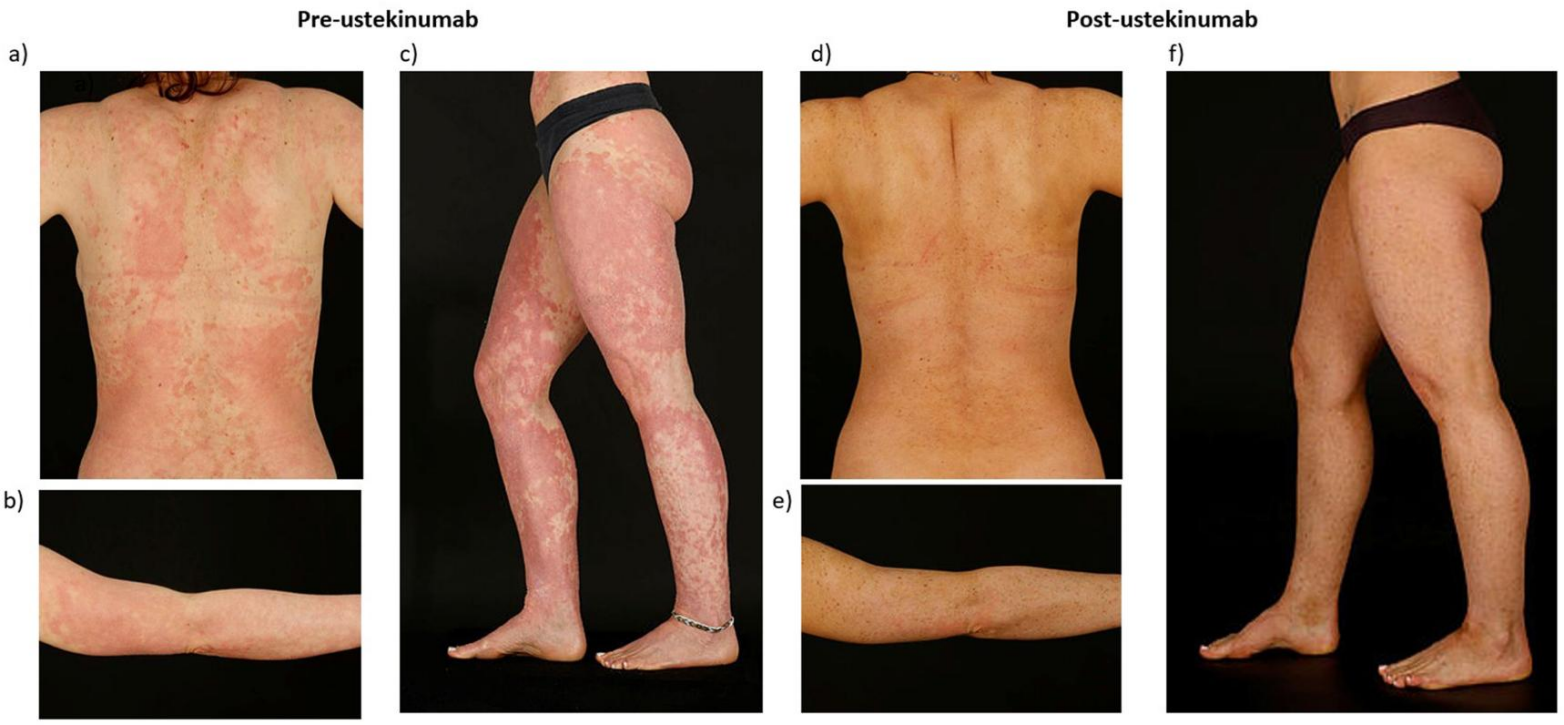
Extent of pityriasis rubra pilaris before (a–c) and after* (d–f) introduction of ustekinumab. Visible reduction in erythema and scaling over whole body, most apparent improvement on the legs. *Patient is wearing self‐tan in the post‐ustekinumab images.

Her skin was very well controlled on acitretin 20 mg daily for many years. Aged 29 years she was referred to the dermatology genetics clinic to explore alternative treatment as the patient was considering trying to conceive in the future. She was switched to isotretinoin 40 mg daily. Blood sampling for genetic testing was performed. One year later, her skin was notably worse with extensive, erythematous, scaly plaques, more confluent on the abdomen and thighs (Figure 1a–c). The working diagnosis was changed to PRP. She commenced ciclosporin 100 mg twice daily (BD), later increased to 150 mg BD. After one year of treatment without adequate control, the multidisciplinary team agreed to commence biologic therapy.

She commenced 12‐weekly subcutaneous ustekinumab 45 mg. Within one month of introduction, there was a significant improvement in skin appearance and her dermatology life quality index (DLQI) score improved from 19 to 5 (Figure 1d–f). This control was maintained after interval dose adjustment to 10‐weekly administration of ustekinumab 45 mg. Her dosing regimen mirrors the literature.^2^ Next generation sequencing (Panel R165, ICHTHY_v1.3_R165_Green)^3^ identified a heterozygous *CARD14* mutation (c.356 T > C p.[Met119Thr]) classified as ‘likely pathogenic’ by the genetics laboratory. Given that this patient's parents are not affected, but have not had genetic testing, it is suspected that this is a de novo mutation.

PRP is a rare, papulosquamous, inflammatory dermatosis. Historically it was divided into five subtypes based upon disease extent, age of onset and expected prognosis, however a sixth HIV‐related PRP has since been recognised.^1^, ^4^ The most common form is type I (classical adult), which carries the best prognosis. Other forms are types II (atypical adult), III (classic juvenile), IV (circumscribed juvenile) and VI (HIV‐associated).^1^ Atypical juvenile (type V) PRP represents 5% of cases and is most associated with familial forms of PRP and presence of *CARD14* mutations.^5^ It is characterised by early onset, protracted disease, with follicular hyperkeratosis, ichthyosiform features and sclerodermatous changes of the hands and feet.^1^, ^4^, ^5^ Our patient has atypical juvenile PRP with diagnosis delayed into adulthood. Earlier genetic sequencing and diagnosis may have enabled a personalised approach to treatment, including exploring other opportunities for family planning.

The term *CARD14* Associated Papulosquamous Eruption (CAPE), first proposed in 2018, describes the spectrum of conditions associated with features of psoriasis, PRP, or both, and *CARD14* mutations.^6^ *CARD14* is highly expressed in the skin and mutant forms are independently associated with psoriasis and PRP.^5^ *CARD14* mutations result in inappropriate NF‐kB and mitogen‐activated protein kinase pathway activations thought to cause CCL20 overexpression, and IL13 and IL17A activation.^5^, ^7^

Ustekinumab is a monoclonal antibody against the IL12 and IL23 p40 subunit and has downstream effects on the NF‐kB pathway, hence is a pathogenesis‐based treatment for PRP.^8^ Good response to ustekinumab has been described in several PRP cases.^2^, ^8^, ^9^ Response to ustekinumab in the literature varies, however is largely successful, with five out of six patients in one case series of patients with CAPE experiencing near complete response to ustekinumab.^6^ Anti‐IL17 agents including ixekizumab^10^, ^11^ and secukinumab^12^ have also been used successfully for the treatment of CAPE.

Due to the rare nature of CAPE, treatment options for PRP are primarily based upon case reports. This patient experienced a dramatic improvement with ustekinumab combined with ciclosporin for PRP associated with a *CARD14* c356 T > C variant. This case augments the literature supporting the use of ustekinumab for atypical juvenile PRP, or CAPE. It highlights the role of genetic testing in dermatological conditions where there is diagnostic uncertainty to enable personalised treatment and improved patient outcomes.

## CONFLICT OF INTEREST STATEMENT

**RMC:** Position funded by Palvella Therapeutics to work on a clinical trial. All unrelated to this work and all funding goes to the university. **EOT:** Research funding: Kamari Pharma, Unilever. Consultancy: Azitra Inc, Palvella Therapeutics and Kamari Pharma; Speaker: Almirall. All unrelated to this work and all funding goes to the university.

## AUTHOR CONTRIBUTIONS

**Rebecca L. McCarthy**: Conceptualization (lead); formal analysis (equal); investigation (equal); methodology (equal); resources (lead); writing – original draft (lead). **Jaimie Oldham**: Conceptualization (supporting); data curation (supporting); investigation (supporting); writing – review & editing (supporting). **Elsa Barbosa**: Conceptualization (supporting); data curation (supporting); investigation (supporting); writing – review & editing (supporting). **Catriona Sinclair**: Data curation (equal); investigation (lead); methodology (equal); writing – review & editing (supporting). **Malvina Cunningham**: Conceptualization (equal); data curation (equal); formal analysis (equal); investigation (lead); methodology (equal); resources (equal); writing – review & editing (equal). **Edel A. O’Toole**: Conceptualization (lead); data curation (equal); investigation (equal); methodology (equal); supervision (lead); writing – review & editing (lead).

## ETHICS STATEMENT

We obtained written informed consent for the use of the patient's anonymised clinical information and images.

## Data Availability

Data sharing is not applicable to this article as no new data were created or analyzed in this study.
